# Computed tomography scanning can monitor the effects of soil medium on root system development: an example of salt stress in corn

**DOI:** 10.3389/fpls.2015.00256

**Published:** 2015-04-28

**Authors:** Sowmyalakshmi Subramanian, Liwen Han, Pierre Dutilleul, Donald L. Smith

**Affiliations:** Department of Plant Science, McGill UniversityMontréal, QC, Canada

**Keywords:** corn (*Zea mays* L.), NaCl salt stress, developing root systems, structural complexity, fractal dimension (FD), root volumes and relative growth rates, root lengths and increments, computed tomography (CT) scanning

## Abstract

Seeds and young seedlings often encounter high soluble salt levels in the upmost soil layers, impeding vigorous growth by affecting root establishment. Computed tomography (CT) scanning used at low X-ray doses can help study root development in such conditions non-destructively, because plants are allowed to grow throughout the experiment. Using a high-resolution Toshiba XVision CT scanner, we studied corn (*Zea mays* L.) root growth under optimal and salt-stressed conditions in 3D and on a weekly basis over 3 weeks. Two groups of three corn plants were grown in the controlled environment of a growth chamber, in mid-sized plastic pots filled with sieved and autoclaved sand. Seedlings were subjected to first CT scanning 1 week after seed planting. Our main research objectives concerning root systems were: (i) to quantify structural complexity from fractal dimensions estimated on skeletal 3-D images built from CT scanning data; (ii) to measure growth from volumes and lengths and the derived relative rates and increments, after isolating primary and secondary roots from the soil medium in CT scanning data; and (iii) to assess differences in complexity and growth per week and over Weeks 1–3 for groups of corn plants. Differences between groups were present from Week 1; starting in Week 2 secondary roots were present and could be isolated, which refined the complexity and growth analyses of root systems. Besides expected Week main effects (*P* < 0.01 or 0.05), Week × Group interaction (*P* < 0.05 or 0.10), and Group main effects were observed. Graphical, quantitative, and statistical analyses of CT scanning data were thus completed at an unprecedented level, and provided new and important insights regarding root system development. Repeated CT scanning is the key to a better understanding of the establishment in the soil medium of crop plants such as corn and the assessment of salt stress effects on developing root systems, in complexity, volume, and length.

## Introduction

Plants encounter various abiotic and biotic stressors during their life cycle. Two of the most prevalent abiotic stressors confronting global agriculture are soil salinity and drought. Salinization is one of the more serious agricultural limitations, especially in the arid and the semi-arid regions of the world. Approximately 20% of the world’s irrigated land yields a third of the global food stocks. At the same time, about 30% of this highly productive land is affected by salinity (Yan, 2008; Xu et al., 2011). Land clearing and irrigation are among the major contributors to salinization of agricultural lands. Their general impact has been reported (Munns, 2005; Rengasamy, 2008), and this is aggravated by a number of factors, including climate, the degree of water deficit, the inherent salt content of soils, topography, and the underlying geology and hydrology (Wiebe et al., 2007).

During the initial development of a seed into a plant, the germinating seed puts forth the radical that subsequently differentiates into the root system, the fundamentals of which determine plant growth and productivity. A plant’s response to salinity is a complex process which affects the plant’s tissue and organ development at various stages of growth. Seed germination under saline conditions cause significant reductions in seed germination percentage, thereby causing uneven stands and reduced yield (Foolad et al., 1999). Sodium chloride (NaCl) is a dominant salt in nature, which at sufficiently high concentrations reduces the ability of plants to take up water (water-deficit effect) and other essential nutrients (ion-excess effect; Munns, 2005; Munns et al., 2006). The uppermost soil layers are generally the site of highest soluble salt accumulation due to evaporation and capillary rise of water, so that seeds and young seedlings are frequently confronted with salinities much too high to allow vigorous growth (Almansouri et al., 2001). However, depending on the soil type and irrigation, or the subsoil salinity independently of the water capillary rise, a saline gradient is usually seen in many saline fields that affect root spread of crops (Wiebe et al., 2007; Rahnama et al., 2011).

Salt stress causes changes in plant growth through (1) osmotically induced water stress, (2) specific ion toxicity due to high concentrations of sodium and chloride, (3) nutrient ion imbalance due to high levels of Na^+^ and Cl^-^, which reduce the uptake of K^+^, NO^-^, and PO_4_^3-^, and (4) increased production of reactive oxygen species (ROS), which damage macromolecules inside plant tissue, all of which result in plant growth reduction (Greenway and Munns, 1980). For instance, salt stress enhances the accumulation of NaCl in chloroplasts of higher plants, which affects their growth rate, and is often associated with a decrease in photosynthetic electron transport activities (Kirst, 1990). Additionally, in higher plants, it inhibits photosystem-II activity (Kao et al., 2003; Parida et al., 2003), which indirectly reflects upon the below-ground biomass, the roots. Simulation of a salinity gradient using a PVC tube with paper roll soaked in salt demonstrated sensitivity to salt for roots of wheat cultivars with regard to branching (Rahnama et al., 2011). Screening of genotypes of wild and domestic barley for salinity stress suggests large variations in response to salt in Petri plate assays. Increasing salt concentration (100–150 mM NaCl) decreased both shoot and root growth in various aspects in barley cultivars (Shelden et al., 2013), suggesting saline soils substantially alter plant metabolic processes (Levitt, 1980).

Corn is the third major cereal crop produced across the globe (FAO, 2014), and is grown under a very wide range of climatic conditions. The seedling structure of the family Poaceae is unique among monocotyledonous plants (Coudert et al., 2010). Specific terms such as scutellar node, coleoptilar node, mesocotyl, and coleorhizae, coined by Tillich in 1977 (Tillich, 1977; Hochholdinger et al., 2004), have been since used to describe these structures. The root system architecture of corn is complex, and was described by Abbe and Stein (1954). During the plant’s life cycle, an embryonic root system and a post-embryonic root system can be distinguished. The embryonic root system consists of a primary root and a number of seminal roots, all of which dominate during the first 2 weeks of seedling development and establishment. It is followed by the early post-embryonic root system in which the primary and seminal roots develop lateral roots 6–7 days after initialization of the embryonic root system. The post-embryonic root system consists of shoot-borne roots formed at the nodes, called the crown roots, 10–14 days after seed germination. Roots developing on the above-ground nodes are called the brace roots. All the lateral roots that developed from the embryonic and post-embryonic systems influence the architecture of the whole system through the branching patterns, including the secondary and higher-order roots; they govern the anchorage as well as the nutrient and water uptake, and are sensitive to environmental factors (Hochholdinger et al., 2004; Lynch, 2013).

Differences in root architecture allow the crops to explore various soil domains at different intensities, in coordination with other environmental factors (Schwinning and Weiner, 1998; Postma and Lynch, 2012). The study of a root system’s architecture is of importance to plant breeders because genetic variation and a suite of quantitative trait loci are involved in its development and functioning (de Dorlodot et al., 2007). The plasticity and dynamics of root system architecture are equally important for the manipulation of crop agronomic traits (Richards, 2008; Zhu et al., 2011), since a proper understanding is required to develop and breed crops for targeted environments (Smet et al., 2012). In root system architecture studies involving field-grown corn, the secondary and higher-order roots that developed after the primary and seminal roots contribute significantly to the total root number and total root length, although the root length of specific orders can vary according to soil types (Wu and Guo, 2014).

Corn is a C4, cross-pollinated, and highly polymorphic crop, with variable salinity tolerance across genotypes. When grown under saline conditions, it can show decreased growth and yield, several of its growth variables being affected at early seedling and growing stages, with the roots being affected the most (Carpici et al., 2009). Nuclear magnetic resonance studies suggest that corn root tips accumulate sodium rapidly (Spickett et al., 1993). Differences in the pattern of root solute potential were observed in corn treated with NaCl as a salt accumulation treatment vs. a salt shock treatment with the administration of 100 mM NaCl as a single dose; the sudden shock caused rapid inhibition of root extension, accompanied by decreased root solute and potential (Rodríguez et al., 1997).

Computed tomography (CT) scanning was originally designed as a medical diagnostic tool in the early 1970s (Kalender, 2000), and is now applied in a variety of non-medical fields, such as archeology, biology, the soil, material and earth sciences, the timber industry, industrial inspections, and aviation security, owing to its non-invasiveness combined with high spatial resolution based on indirect matter density measurement (van Kaick and Delorme, 2005). Very importantly, the low dose of X-rays used in studies involving a medical-type CT scanner, compared to industrial CT scanners which use much higher doses, has been proved to be adequate to study developing plant structures (Dutilleul et al., 2005, 2008; Lontoc-Roy et al., 2005; Han et al., 2008, 2009). A recent study on rice root variables and the associated microbial communities suggests that there were no significant differences between non-CT scanned and CT scanned samples (Zappala et al., 2013).

The use of CT scanning technology with plants was initiated in the late 1990s in an approach alternative to destructive characterization and rhizotron-based observation of root branching patterns. Thus, Heeraman et al. (1997) were able to visualize, non-invasively in 3D, 0.8 cm of bush bean roots (*Phaseolus vulgaris*), but much remained to be done in terms of graphical, quantitative, and statistical analyses of plant CT scanning data. The comparison of destructive vs. CT scanning-based characterization of a root system was among the research questions initially investigated. Actually, a reliable comparison between the two procedures, applied for the same root system, is difficult and even practically impossible, as the CT scanning must precede the destructive characterization; accordingly, the two will never be applied at exactly the same time and in exactly the same experimental conditions (e.g., root moisture), as destructive characterization usually involves root washing. Some studies (chickpea: Perret et al., 2007; cereals: Flavel et al., 2012) suggest an underestimation of total root length with CT scanning, which would mean the incompleteness of root isolation by CT scanning has been larger than the root loss by soil separation and washing in destructive characterization. Nevertheless, Lontoc-Roy et al. (2006) clearly showed that the 3-D image of a corn root system reconstructed from CT scanning data collected in an appropriate soil-moisture condition for the type of soil medium used provides a more reliable basis for fractal analysis and the estimation of a fractal dimension (FD) as measure of structural complexity, than 2-D photographs of the same root system extracted from the soil and analyzed using the fractal analysis module of the WinRhizo software (Regent Instruments Inc., Québec City, QC, Canada). Definitely, CT scanning technology helps capture details of root systems, such as lateral root growth and its orientation, the variables of which cannot be studied using a destructive method (Perret et al., 2007; Han et al., 2008).

Therefore, we have used a high-resolution X-ray CT scanner to study the architecture of developing root systems of corn variety 19K19 under optimal and salt-stressed conditions, at an unprecedented level of graphical, quantitative, and statistical analyses. Our main research objectives concerning root systems were: (i) to quantify structural complexity from FDs estimated on skeletal 3-D images built from CT scanning data; (ii) to measure growth from volumes and lengths and the derived relative rates and increments, after isolating primary and secondary roots from the soil medium in CT scanning data; and (iii) to assess differences in complexity and growth per week and over Weeks 1–3 for groups of corn plants. Hence, our main biological research objective is to further our knowledge and understanding of the below-ground response of corn to salinity stress at the initial stages of plant development.

## Materials and Methods

### Plant Material, Soil Preparation, and Salt Stress Imposition

Seeds from one of the highest yielding organic corn varieties, 19K19 Blue River, procured from Doug Shirray Seeds and Ag supplies (Tavistock, ON, Canada), were used in our experiments since these are among the most easily available untreated corn seeds. The growth medium was sand, which was sieved to 2 mm to homogenize the rooting media, autoclaved, and kept dry for at least 1 week before potting. In black plastic pots with an 18-cm diameter at the top, a 0.1-mm side-wall thickness, and 17-cm total height (Classic®400; Plant Products Co. Ltd., Laval, QC, Canada), filled with sieved and autoclaved sand, one corn seed was placed in the center at a depth of 2.5 cm. Three such pots were prepared for control and three more for the salt stress. Thereafter, the pots were uniformly watered, and the seedlings were allowed to emerge. One day after emergence, the pots were given ½ Hoagland solution (Hoagland and Arnon, 1950) for control and ½ Hoagland + s100 mM NaCl for salt stress as a one-step salt shock. According to our results from other experiments with this cultivar of corn, 100 mM NaCl imposition was a salt stress for which this cultivar expressed a response.

The plants were grown in a growth chamber (Conviron Model No. PGR15, Controlled Environments Ltd, Winnipeg, MB, Canada), set at 25 ± 2°C (day temperature) and 22 ± 2°C (night temperature), with a photoperiod of 14/10 h day/night cycle, 60–70% relative humidity and photosynthetic irradiance of 350–400 μE m^-2^ s^-1^. Subsequently, the pots were given ½ Hoagland once a week. The watering was scheduled so as to reduce moisture content of the sand rooting medium at the times of CT scanning, thus following Lontoc-Roy et al. (2006); this way of proceeding has the positive effect of increasing the contrast between roots and soil (in the case of sand) in the CT images, and consequently improves the CT scanning data analyses.

### CT Scanning Configuration, Data Collection and Processing, and Skeletal Root Image Construction

The CT Scanning Laboratory for agricultural and environmental research on Macdonald Campus of McGill University (Ste-Anne-de-Bellevue, QC, Canada) contains a Toshiba XVision high-resolution CT scanner (Toshiba Corporation, Medical Systems Division, Tokyo, Japan). Our experimental corn seedlings were CT scanned in this facility, once a week for 3 weeks; more specifically, it is the plant–soil materials in the pots that were CT scanned. The first sessions of CT scanning (May 7–8, 2012) corresponded to 7 or 8 days after the seeds were planted in the pots. It was not possible to CT scan all six plants in 1 day, so the order for CT scanning was randomized the first week and repeated the next 2 weeks; on each day, one plant from one group and two plants from the other group were CT scanned; between CT scanning sessions and until completion of the experiment, the potted corn seedlings were maintained in the growth chamber. Each potted plant was positioned horizontally on the couch of the CT scanner, and entered the gantry ‘stem first,’ for a top-to-bottom CT scanning of its content. Earlier tests and previous experiments suggest this approach to be better for tracing the roots embedded in the soil during the procedure of CT scanning data analysis (Lontoc-Roy et al., 2005; Han et al., 2008). The helical CT scanning mode was chosen with an image reconstruction interval length of 0.4 mm along the *Z* axis. Other configuration parameter values were based on the experimental conditions, such as the density of the rooting medium used and the size of the object to be scanned. Hence, the field of view was set at SS (18-cm diameter), with no zoom factor (value of 1.0); the tube voltage, at 120 kV; and the tube current, at 150 mA. Every CT scanning session for each root system comprized of 300 cross-sectional CT images covering a depth of 12 cm.

The raw CT scanning data were obtained using the FC70 function, and consisted in CT numbers (CTN), expressed in Hounsfield units (HU). By definition, a CTN is an average relative measure of the density for a pixel in a CT image or for the corresponding volume (voxel) with equal lengths and a width as third dimension (0.35 mm × 0.35 mm × 0.4 mm in this study):

(1) $$CTN(HU)=\frac{\mu_{object}-\mu_{water}}{\mu_{object}-\mu_{air}}\,\;times\,\;1000\,$$

where μ*_object_* = mean value of the linear attenuation coefficient for the voxel; μ*_water_* = linear attenuation coefficient of pure water; and μ*_air_* = linear attenuation coefficient of a standardized air sample. The CTN scale is linear and is centered at 0 to correspond to water, and the CTN for air is calibrated to -1000 HU. Hence, densities greater or less than water correspond to positive and negative CTNs, respectively.

The raw CTN data thus collected (300 512 × 512 matrices of CTNs) were first displayed on the CT console and further processed on a 4-core Dell i3 computer. Using MATLAB 2013b and 2014a (The MathWorks Inc., Natick, MA, USA), the data for a given corn seedling CT scanned in a given week were converted into an internal 3-D array, and scrutinized with a Graphical Unit Interface application program created to view the cross-sectional CT images and produce digitally on computer the 3-D architecture of the first-order roots and as many secondary roots of the system as possible. The construction of skeletal 3-D images of the corn root systems was performed in this environment.

### Fractal Dimension Estimation

As already noted by Foroutan-pour et al. (1999a) for fractal analysis from photographs of branching patterns of soybean seedlings, it is very important to skeletonize the image of the structure of interest, whether it is a shoot branching pattern or a root system, in order to perform an unbiased estimation of the FD; by “skeletonization,” we mean reducing the thickness of a branch or a root to 1 pixel in the 2-D image, or 1 voxel in 3D. Based on the methodological results of Foroutan-pour et al. (1999b), it is also very important not to use the full range of box sizes in the box-counting procedure of FD estimation in 2D, or the full range of cube sidelengths in the cube-counting procedure in 3D; instead, it is recommended to use, in 3D, a subset of cube sidelengths that does not contain the smallest and largest lengths. This follows the approach of Han et al. (2008), who analyzed 3-D skeletal images of potato root systems; we have followed and applied their FD estimation procedure.

Among the nine cube sidelengths available in our study, after trying all possible subsets including 3, 4, or 5 middle length values, we retained the FD estimates obtained after discarding the three smallest and three largest cube sidelengths, because this was found to provide the highest *R*^2^-values in the log–log plot

(2) log⁡[N(s)]=k+FD⁢ log⁡(1/s)

where log(^.^) is the natural logarithm, *N*(*s*) denotes the number of cubes with sidelength *s* intersecting the skeletal root system, and the straight line is fitted by ordinary least squares.

### Root Volumes and Relative Growth Rates

**FIGURE 1:**
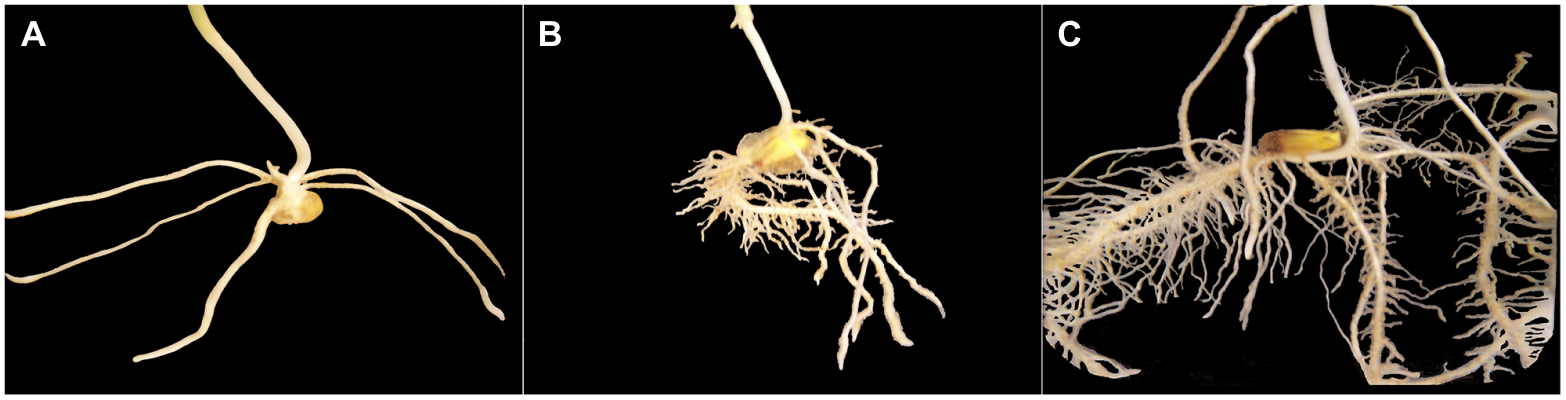
**Excerpts of photographs of corn root systems immerged in a container filled with water; only the root system appearing in a photograph is shown on a black background.** The three corn seedlings grown for this illustration (variety 19K19 Blue River, same rooting medium, Hoagland nutrition solution) had reached stages of development comparable to those of Weeks 1–3 (from **A–C**) for the control corn seedlings in our experiment.

In preparation of our experiment, we had made preliminary tests using root systems of corn seedlings other than the experimental ones but of same variety, grown in same rooting medium with same Hoagland nutrition solution as the control group. One of our goals in doing this was to photograph corn root systems at same developmental stages (1, 2, and 3 weeks after seed planting) immerged in a container filled with water (**Figure 1**), for later comparison with 3-D images constructed from CT scanning data. Another goal was to make manual measurement of thickness on those corn roots once washed, so the procedure described below was applied with confidence to construct non-skeletal 3-D images of the experimental corn root systems from CT scanning data. Our procedure also takes into account the difference in size between primary and secondary roots and the spatial resolution of the CT scanning data collected (voxel dimensions: 0.35 mm × 0.35 mm × 0.4 mm).

The skeletal primary roots were ‘grown’ by up to four layers (one layer a time), in 3D, if the CT numbers of the neighboring voxels did not exceed 850 HU; for secondary roots, the threshold used for CT numbers was 950 HU and the maximum number of layers added to the skeleton was two. From the primary and secondary roots thus ‘grown,’ three types of root volumes were calculated for each of the six experimental corn seedlings in each of the 3 weeks: for lower roots alone, for upper roots alone, and for lower and upper roots combined. The stem and seed were not included in the evaluation of root volumes. Relative growth rates between successive Weeks *t* and *t*+1 were calculated accordingly from the estimated root volumes (denoted Root vol_*t* and Root vol_*t*+1 below):

(3) Relative⁢ growth⁢ rate −Week⁢ t+1,Week⁢  t=(Root⁢ vol−t+1−Root⁢ vol−t)/Root⁢ vol−t

### Root Lengths and Length Increments

MATLAB, in combination with the image analysis toolset ImageJ (National Institutes of Health, Bethesda, MD, USA), were used for root length measures, in the following sequence of steps and operations. First, the 3-D array for the skeleton of root system of a given corn seedling CT scanned in a given week was loaded in MATLAB. Using the MATLAB function *imwrite*, each slice, out of 300 available per corn seedling per week, was then exported as an 8-bit gray image with a given format (i.e., BMP), into a designated folder. An image stack was then built from all the images after these were imported into ImageJ. The corresponding 3-D image was skeletonized with the ImageJ skeletonization procedure, and a customized 3-D analysis plugin (https://github.com/fiji/AnalyzeSkeleton/) was used to perform the root length measurements. Finally, the output was summarized to obtain total lengths of lower and upper roots separately. Say Root length_*t* and Root length_*t*+1 are measures of total root length for a given corn seedling in Weeks *t* and *t*+1; the corresponding increment was then calculated as

(4) $$Root\,\;lenth\,\;increment_{-}Week\,\;t+1,\;Week\,\;\;t=Root\,\;\;lenth_{-}t+1-Root\,\;lenth_{-}t$$

### Statistical Analyses

Sample means per group (Control, optimal vs. Salt, salt stress) and the corresponding SEs were computed and plotted on a weekly basis. Furthermore, analysis of variances (ANOVAs) for temporal repeated measures (ANOVARs; Crowder and Hand, 1990; Dutilleul, 2011) were performed on the data tables of FD estimates, root volumes and lengths (1 weekly observation = 1 temporal measure), as well as on those of fractal dimension ratios (FDR) between the initial FD estimate and the next two, relative growth rates (Eq. 3), and root length increments (Eq. 4). Univariate ANOVARs using the sample variance–covariance matrix in the modified *F*-ratio tests were carried out in SAS 9.3 PROC GLM, option REPEATED (SAS Institute Inc., Cary, NC, USA), because the small sample sizes did not allow a modeling of the variance–covariance structure in a mixed-model approach. For the same reason, we considered three significance levels, namely 0.01, 0.05, and 0.10, in our hypothesis testing. Differences among treatments were only considered meaningful when occurring at one of these significance levels, and when these are discussed, the *P*-value is bolded. The between-group homogeneity of variance was tested, and rejected only once (i.e., for volume of lower roots in Week 1) in 15 tests, with no consequence for our results.

## Results

Differences in structural complexity and space occupancy of the developing root systems of corn seedlings, grown in sand under optimal condition vs. salt stress, were investigated in 3D. Structural complexity was measured through FDs of skeletal 3-D images, and space occupancy, through root volumes. Between-group differences were assessed absolutely and relatively in and over the 3 weeks of the experiment. Results are presented below and in tables and figures.

### Three-Dimensional Image Analyses

**FIGURE 2:**
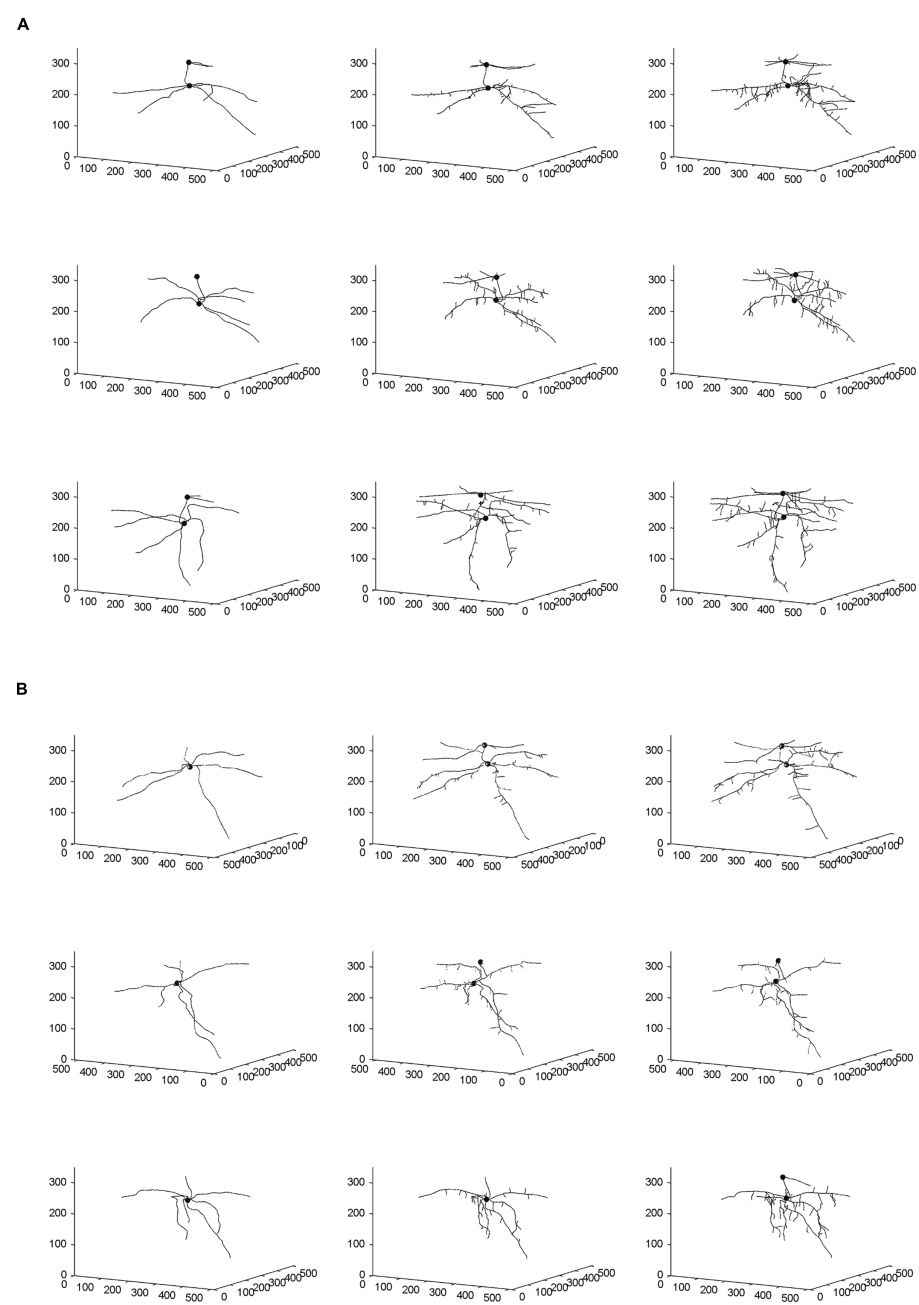
**Skeletal 3-D images of corn root systems for **(A)** optimal and **(B)** salt-stress treatments, as constructed from computed tomography (CT) scanning data collected on a weekly basis over 3 weeks (i.e., Weeks 1–3 from left to right) for the three individual plants in each treatment group.** In each panel, the horizontal plane in the 3-D rendering represents the X-Y plane in CT scanning terminology; this plane is perpendicular to the couch of the CT scanner. The small filled spheres locate the connection points of the primary upper and lower roots to the below-ground part of the stem.

**FIGURE 3:**
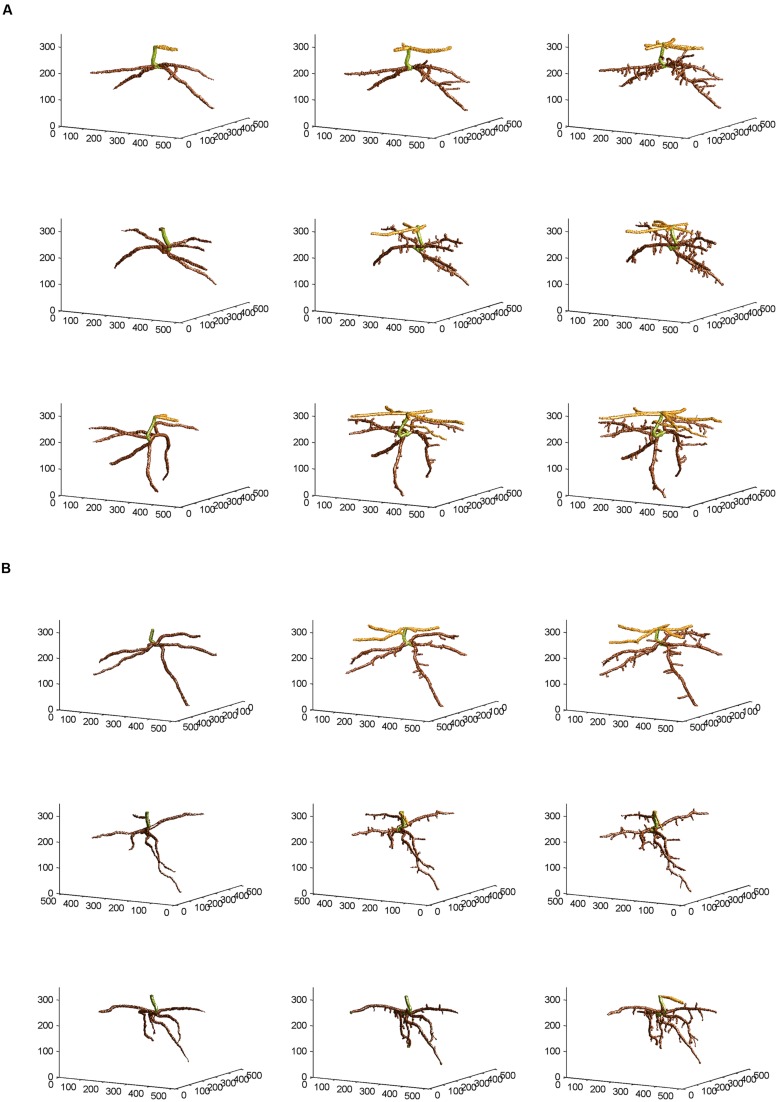
**Non-skeletal 3-D images of the corn root systems depicted in skeletal form in **Figures 2A,B** for **(A)** optimal and **(B)** salt-stress treatments, respectively; the below-ground part of the stem was false-colored in green, the upper roots in light brown, and the lower roots in dark brown, with a slight glossy effect.** See text for the procedures of expansion from the skeletal images and attachment of corn root volumes to the skeletons, using the CT scanning data collected and applying appropriate thresholds to them.

From the skeletal 3-D images of root systems constructed from the CT scanning data collected on a weekly basis for individual plants in the control and salt-stressed groups (**Figures 2A,B**), it is clear that germinated seeds of the control group in Week 1 (left panels of **Figure 2A**) show the development of embryonic roots with two subsets of roots called “upper roots” and “lower roots” here, whereas the onset of upper roots is delayed in the salt-stressed plants and upper roots in this group are present and visible only from Week 2 on (middle and right panels of **Figure 2B**). Also, the branching patterns of root systems in Weeks 2 and 3 show less prominent lateral branching in salt-stressed plants, compared to control plants. **Figures 3A,B** show the root volumes that it has been possible to isolate from the plant–soil CT scanning data, by expansion from the initial skeletons of root systems. Graphically, all of this strongly suggests that during the initial stages of plant growth, the salt abiotic stressor (100 mM NaCl) has negative effects on the development of the root system, and weakens the below-ground establishment of the corresponding seedling relative to one grown under optimal conditions.

### Fractal Analyses and ANOVARs on FD Estimates

**FIGURE 4:**
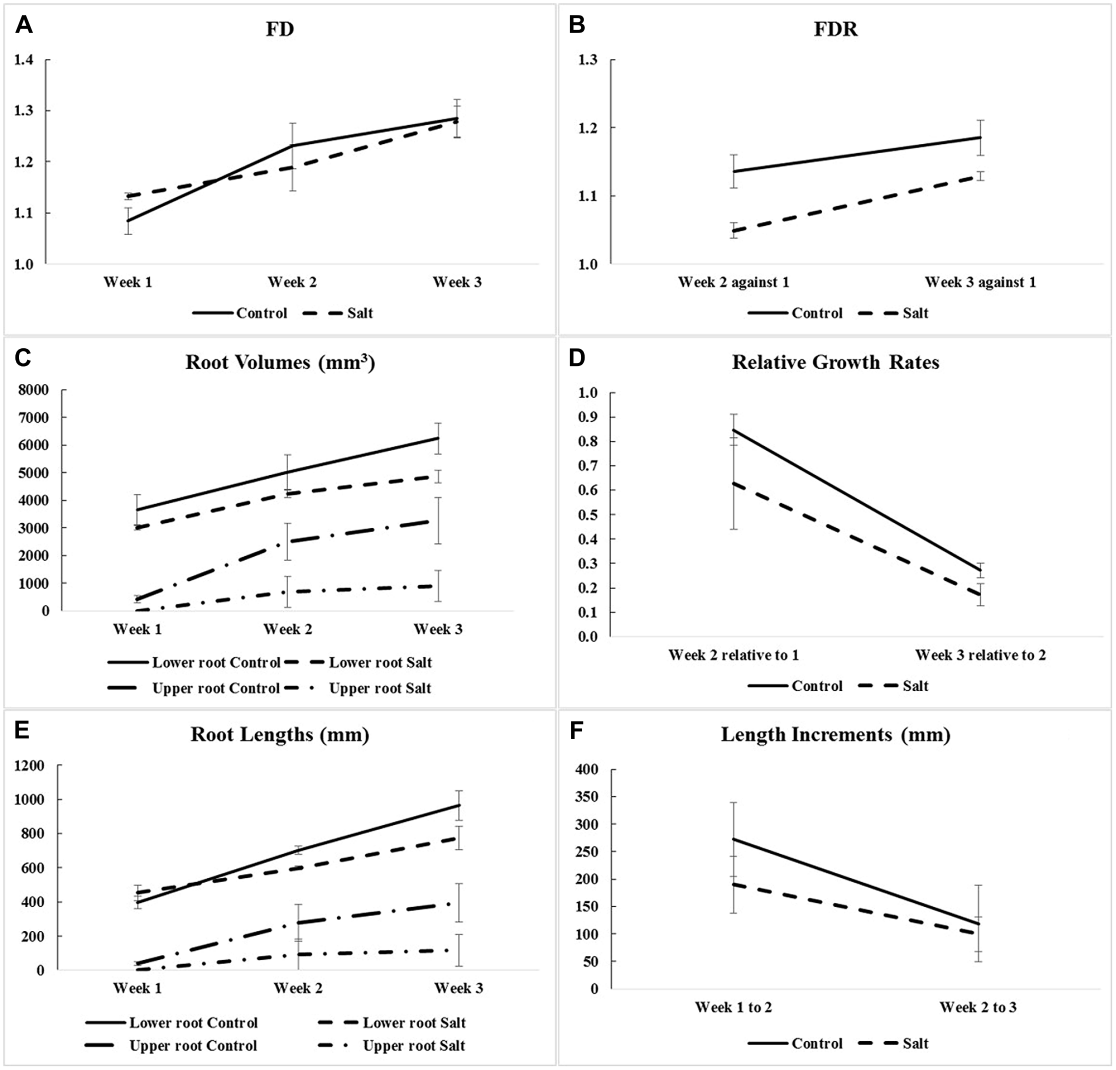
**Means and SE of corn root system traits measured for optimal (Control) and salt-stress (Salt) treatment groups: **(A)** fractal dimensions (FD) in Weeks 1–3 (only for lower-root systems; see **Table 1**); **(B)** fractal dimension ratios (FDR; i.e., against Week 1); **(C)** root volumes in Weeks 1–3 (for lower- and upper-root systems); **(D)** relative growth rates (i.e., relative to the previous week and calculated using volumes of lower and upper roots combined); **(E)** root lengths in Weeks 1–3 (for lower- and upper-root systems); and **(F)** length increments (i.e., between successive weeks and calculated using root lengths of lower- and upper-root systems combined)**.

Fractal analysis was restricted to lower roots, in the absence of upper roots for all three salt-stressed plants in Week 1 and for one of them in Week 2. Combining upper roots, when present, with lower roots in the same fractal analysis would not be recommended anyway, because the two types of roots form distinct systems by themselves (**Figures 2** and **3**). Overall, the observed FD values are low to moderately high, that is, above 1.0 (which classically corresponds to a straight line) but well below 2.0 (a plane), and actually below 1.5; this could be expected for young plant material such as root systems of corn seedlings only a few weeks old. On a weekly basis (mean ± SE), FD estimates ranged from 1.084 ± 0.026 (Week 1) to 1.284 ± 0.037 (Week 3) in the control group vs. from 1.132 ± 0.006 (Week 1) to 1.279 ± 0.030 (Week 3) in the salt-stressed group, with 1.231 ± 0.044 vs. 1.188 ± 0.045 in Week 2 for the two groups, respectively. The crossing of curves between the two groups from Week 1–2 in **Figure 4A**, which is the result of a higher FD mean value in Week 1 for the salt-stressed group and a higher FD mean value in Week 2 for the control group, led us to analyze the ratios of FD estimates in Weeks 2 and 3 relative to the corresponding FD estimate in Week 1 (**Figure 4B**), and eventually, perform two ANOVARs instead of one.

The ANOVARs indicate non-significant (*P* ≥ 0.10) between-group differences in the FD mean values for the 3 weeks taken separately as well as in the two FDR mean values, considering one ratio at a time. However, on average over the 3 weeks (see Between-subject effects in **Table 1**), the between-group difference in the FD overall mean value is significant (*P* < 0.10). Additionally, the Week main effects (see Within-subject effects in **Table 1**) are highly significant (*P* < 0.01) for the FDs, and are only significant (*P* < 0.10) for the FDR. To be complete, the difference in the FD mean value is significant (*P* < 0.05) between Weeks 1 and 2, and highly significant (*P* < 0.01) between Weeks 1 and 3.

**Table 1:** Repeated measures analysis of variance (ANOVAR) results for fractal dimensions (FD) and fractal dimension ratios (FDR) for corn lower-root systems.

Source	*df*	FD	Source	*df*	FDR
		***P*-value**			***P*-value**

Group^†^ (in Week 1)	1	0.1435	Group^†^ (Week 2/Week 1)	1	0.1248
Group^†^ (in Week 2)	1	0.5397	Group^†^ (Week 3/Week 1)	1	0.1693
Group^†^ (in Week 3)	1	0.9118			
**Between-subject effects**
Group	1	0.9967		1	**0.0654**
Error	4			4	
**Within-subject effects**
Week	2	**0.0004**		1	**0.0779**
Week × Group	2	0.2463		1	0.6161
Error (Week)	8			4	

^†^The Error df is 4 (df: number of degrees of freedom).

### ANOVARs on Root Volumes and Relative Growth Rates

As shown in **Figure 4C**, the weekly means of lower-root volume for the control group are systematically above those for the salt-stressed group; furthermore, this is also the case for the weekly means of upper-root volume, due to the delayed onset of upper roots for salt-stressed plants (see “Three-Dimensional Image Analyses” and **Figures 2** and **3**). Relative growth rates of lower-root volume between Weeks 1 and 2 are almost equal in the two experimental groups; for all the other relative growth rates that we considered, the mean value for the control group is greater than that for the salt-stressed group, with a difference of 10% or more (**Figure 4D**).

Aside from the obvious difference in means between the two groups for the upper-root volume in Week 1, the ANOVAR performed on root volumes (**Table 2**) provides: (i) significant (*P* < 0.10) between-group differences for the three types of root volumes considered (lower roots alone, upper roots alone, lower and upper roots combined) in Week 3; (ii) significant (*P* < 0.10) Group main effects (Between-subject effects) for the upper-root volume averaged over Weeks 2 and 3; (iii) highly significant (*P* < 0.01) Week main effects (Within-subject effects) for the three types of root volumes; and (iv) a significant (*P* < 0.10) or highly significant (*P* < 0.01) Week-by-Group interaction, indicating an increasing difference in weekly means between groups from the first or second week to the last. As for relative growth rates (**Table 3**), the ANOVAR only found highly significant (*P* < 0.01) Time main effects (Within-subject effects) for the upper-root volume and the lower and upper-root volumes combined (i.e., no significant Week-by-Group interaction); thus, relatively, space occupancy by the root systems increased at the same pace in the two groups of corn seedlings. To be complete, the ANOVA found a significant (*P* < 0.10) difference between groups in their mean relative growth rates for upper-root volume from Week 2 to Week 3.

**Table 2:** Repeated measures analysis of variance results for volumes of corn lower- and upper-root systems and the two combined.

Source	*df*	Lower roots	Upper roots	Lower and upper roots combined
		***P*-value**		

Group^†^ (in Week 1)	1	0.3290^§^	N/A^‡^	0.1822
Group^†^ (in Week 2)	1	0.2849	0.1073	0.1494
Group^†^ (in Week 3)	1	**0.0870**	**0.0800**	**0.0803**
**Between-subject effects**
Group	1	0.1956	**0.0908**	0.1136
Error	4			
**Within-subject effects**
Week	2^‡^	**<0.0001**	**0.0093**	**0.0016**
Week × Group	2	**0.0041**	**0.0543**	**0.0759**
Error (Week)	8			

^†^Except for N/A, the Error df is 4 (df: number of degrees of freedom).

^‡^Corn plants of the salt-stressed group had no upper root in Week 1. It follows: (i) the non-relevance of performing a statistical test to compare groups for their upper-root volumes in Week 1; (ii) within-subject effects with 1, 1, and 4 df instead of 2, 2, and 8 df, due to the inclusion of 2 weeks (Weeks 2 and 3) instead of the 3 weeks in the ANOVAR for upper-root volume.

^§^ 0.3776 if a *t*-test with an effective number of df is used, to adjust for the sole case of significant (*P* < 0.05) between-group heterogeneity of the variance in our study.

**Table 3:** Repeated measures analysis of variance results for relative growth rates derived from volumes for corn lower- and upper-root systems and the two combined.

Source	*df*	Lower roots	*df*	Upper roots	*df*	Lower and upper roots combined
				***P*-value**		

Group^†^ (Weeks 1–2)	1	0.7879	N/A^‡^	N/A	1	0.4178
Group^†^ (Weeks 2–3)	1	0.1252	1^‡^	**0.0754**	1	0.1449
**Between-subject effects**
Group	1	0.3040			1	0.2251
Error	4				4	
**Within-subject effects**
Time	1	**0.0080**			1	**0.0121**
Time × Group	1	0.1800			1	0.7376
Error (Time)	4				4	

^†^Except for N/A, the Error df is 4 (df: number of degrees of freedom).

^‡^Corn plants of the salt-stressed group had no upper root in Week 1, so relative growth rates based on upper-root volume could be calculated only from Weeks 2–3 for them, limiting the comparison between groups to a classical type of ANOVA (i.e., without repeated measures).

### ANOVARs on Root Lengths and Length Increments

Graphically, the weekly means of lower- and upper-root lengths and the corresponding increments varied over time and differed between the control and salt-stressed groups, in a way similar to the weekly means of lower- and upper-root volumes and the relative growth rates (see **Figures 4E,F** vs. **Figures 4C,D**). Quantitatively and statistically, a smaller number of significant effects were found for lengths than for volumes, with two significant (*P* < 0.10) effects per type of root when analyzed separately, and combined; nevertheless, five of the six Within-subject effects maintained their statistical significance. On length increments, significant differences between groups are found for lower roots (Weeks 1–2, *P* < 0.10) and upper roots (Weeks 2–3, *P* < 0.01).

## Discussion

### Experimental Approach

Traditionally, studies of root system architecture under laboratory conditions mostly use platforms such as WinRhizo that can help generate data for plants grown in solid medium. For example, a study conducted on a number of *Arabidopsis* accessions, using EZ-Rhizo to screen for root system architecture related to a quantitative trait locus, suggests natural variations across the accessions (Armengaud et al., 2009). Although such an approach is non-invasive, plants grown in Petri plates for such analyses have a critical time frame of study, since the enclosed environment can eventually lead to stressful conditions for plant growth within the Petri plates. Other techniques to screen root system architecture, such as the use of PVC pipes (Shelden et al., 2013) and gel chambers (Bengough et al., 2004) for fast screening of seedlings, or of a Rhizobox or Rhizotron facility (with PVC boxes of various sizes) to study growth of visible roots along the transparent sides of a box, are also useful for short-term studies (Neumann et al., 2009). However, these studies are restricted to 2-D scanning and cannot predict the 3-D growth of roots.

Our study is one of a few in which root systems and their surrounding soil medium have been repeatedly CT scanned (e.g., Lontoc-Roy et al., 2005; Han et al., 2008, 2009). Repeated CT scanning of plant structures in successive stages has multiple advantages, including those of following the development of the same structure over time and capturing the changes. It also has constraints and limits, starting with the use of low X-ray doses because a root system is living plant material. As pointed out by Dutilleul et al. (2005), the radiation output of a CT scanner increases strongly with tube voltage, but it is the product of tube current (mA) by scan time (s) by number of scans that actually represents the radiation level delivered during exposure time. Helical scanning reduces exposure time substantially, while allowing the reconstruction of several images from CT scanning data acquired in one rotation around the sample installed on the couch. In root system studies involving CT scanning, since the plant structure of interest is generally surrounded by a soil medium (i.e., an exception is provided by hydroponic culture) it is equally important that the X-ray dose be sufficiently high to allow radiation to penetrate the soil column throughout. In fact, CT numbers are function of X-ray attenuation coefficients (Eq. 1), and too-low doses would result in imprecise CT numbers for the part of the CT scanned volume near the center of the column in the case of a soil as dense as sand. All in all, some balance must be found, such as the use of 120 kV as tube voltage and 150 mA as tube current for plastic pots with an 18-cm top diameter in our study. Working with smaller containers may be an option to consider depending on the plant species, but when chosen, this option can be at the expense of the last stage up to which the development of root systems can be followed appropriately, since root tips might reach the edges of small containers more rapidly which would alter root growth.

The soil type × moisture content combination is another very important factor to take into account in the repeated CT scanning for underground plant structures. Lontoc-Roy et al. (2006) have shown that sieved homogeneous sand allows a better isolation of corn roots from the plant–soil CT scanning data when it is relatively dry, than when it is water-saturated at the time of CT scanning; for loamy sand (by mass: 78.0% sand, 14.4% silt, and 7.6% clay), it is the contrary. Furthermore, it is recommended that soil moisture content be as much as possible the same at all the times of CT scanning for all the plants, to keep as much as possible the same plant–soil contrasts in the CT scanning data and images and obtain comparable results; this was achieved by watering the corn seedlings after CT scanning, leaving the sand dry before CT scanning, in our experiment. Otherwise, some data transformation can accommodate the situation (Han et al., 2008, 2009). The constraints above are important, but are readily eclipsed by the tremendous insight gained by the follow-up made possible week after week, thanks to the repeated CT scanning, on developing root systems and their non-destructive characterization, with no such extra variability as the one that would be introduced if different seedlings were used for analysis at different times. Of course, as discussed in Subsection “Analytical Aspects,” the repeated-measures nature of FDs and root volumes, for example, must be incorporated in the statistical tests, for them to be valid, in a study like ours.

### Analytical Aspects

Three types of analytical aspects are discussed here: (i) graphical, in relation to spatial resolution, and (ii) statistical, concerning (ii.1) the recommended assessment procedure for effects involving time, and (ii.2) the question of power of the tests concerning treatment effects (e.g., optimal vs. salt stress). Our choice of CT scanning configuration parameter values (field of view: SS or 18 cm, zoom factor: 1.0; see Section “Materials and Methods”) provided a spatial resolution sufficiently fine to isolate from plant–soil CT scanning data and reconstruct all primary roots and portions of secondary roots for the corn seedlings which were 1, 2, and 3 weeks old in our experiment; the voxel dimensions were 0.35 mm × 0.35 mm × 0.4 mm. This would be coarse for smaller root systems, like wheat and rice (Flavel et al., 2012; Zappala et al., 2013); in these two root CT scanning studies, the voxel dimensions were, in fact, smaller. Thus, everything is relative; for a high-resolution CT scanner such as the Toshiba XVision, the scale of observation is in dm and the scale of resolution is 0.1–1.0 mm, while for a ultra-high-resolution CT scanner, they are in cm and 0.01–0.1 mm, respectively (see **Table 1** in Ketcham and Carlson, 2001).

The repeated-measures nature of the characteristics (e.g., FDs, root volumes) derived from 3-D images of reconstructed plant structures in a repeated CT scanning study like ours has implications. The presence of temporal heterogeneity of variance and temporal autocorrelation, two common properties of temporal repeated measures, contradicts the ANOVA assumptions of homogeneity of variance and independence, and the classical ANOVA *F* tests may be invalid. A multiplicative factor (called “Box’s epsilon”; Crowder and Hand, 1990; Dutilleul, 2011) is then applied to the numbers of degrees of freedom of the *F* distribution of the ANOVA test statistic, providing the ANOVAR *F* tests. This generally results in an increase of the *P*-values, thus decreasing the statistical significance of the effects related to the temporal repeated measures (e.g., Week and Week × Group). The modification was slight with three temporal repeated measures in our case, because autocorrelation and heteroscedasticity were then weak, and it is never required with only two repeated measures (i.e., our FDR and relative growth rates).

Now that the experimental approach is well established (see Subsection “Experimental Approach”), the CT scanning of larger numbers of plants per day can be envisaged. Besides a better representation of the treatment effect in time, larger sample sizes (i.e., numbers of individuals in a group) will enhance the power of the statistical tests, meaning that existing effects will be detected more often. From the results reported in **Tables 1–3** and calculations of degrees of freedom made aside, it can be seen that with a few more units in each group, most of the *P*-values between 0.10 and 0.20 in **Tables 1–3** could become smaller than 0.10 or even 0.05, all other things being the same. This would mean a larger number of significant (*P* < 0.10 or *P* < 0.05) effects, especially those related to the treatment (e.g., salt stress), with greater insight into differences in structural complexity and space occupancy of the developing root systems. That said, the number of significant (*P* < 0.01, 0.05, or 0.10) treatment or time-related effects found with three plants per group is remarkable and very encouraging for larger experimentation.

### Biological Significance

Corn salinity tolerance studies have most often focused on the physiological, biochemical, phytohormone, transcriptional, and proteomic responses and in comparison with model plants *Arabidopsis*, rice, and tomato. Salinity responses vary between and within plant organs, growth stages, and are genotype-specific. Normally, roots can differentiate between wet surroundings and air pockets in its environment, and the collective response is referred to as hydropatterning. The immediate environment of the root dictates root hair patterning, positioning, and development of aerenchyma and distribution of anthocyanins and auxin, and is independent of abscisic acid signaling. In *Arabidopsis*, genes such as TRYPTOPHAN AMINOTRANSFERASE OF *ARABIDOPSIS* 1 and PIN-FORMED3 are necessary to control auxin to induce lateral root formation under high water availability. This also dictates the position where the lateral root founder cells need to be positioned and activated (Baoa et al., 2014). However, when a 100-mM NaCl stress is imposed in corn, a 10-fold increase in abscisic acid (ABA) accumulation in roots, as compared to a onefold increase in shoots, has been observed (Jia et al., 2002). Salt-resistant hybrid SR03 was found to have increased indole-butyric acid (IBA) and ABA levels in the shoots, while the roots maintained increased indole-acetic acid (IAA) levels upon 100-mM NaCl stress (Zörb et al., 2013). Quantitative differences in responses to salt stress were observed in salt-sensitive corn cultivar Trihybrid 321 and salt-tolerant cultivar Giza 2. Salt stress decreased fresh weight, dry weight, and relative growth rates of both shoots and roots. An increased accumulation of Na^+^ and Cl^-^ and a decrease in K^+^ and Ca^2+^ were observed in both shoots and roots in cultivar Giza 2 (Mansour et al., 2005). A study on the root growth direction of *Arabidopsis* to salt stress suggests that the roots encountering salt stress have reduced gravity response, and this seems to be modulated by the quantity of salt present in its environment (Sun et al., 2008). The phenomenon of decreased root growth is evident in our study, graphically (**Figures 2** and **3**) as well as quantitatively (**Figure 4**), and statistically (**Tables 1–5**). Salinity stress has an early effect during corn seedling establishment that is more pronounced in the volume aspects than in the structural complexity of the root system.

**Table 4:** Repeated measures analysis of variance results for length measures for corn lower- and upper-root systems and the two combined.

Source	*df*	Lower roots	Upper roots	Lower and upper roots combined
		***P*-value**		

Group^†^ (in Week 1)	1	0.3828	N/A^‡^	0.7720
Group^†^ (in Week 2)	1	**0.0180**	0.2560	0.1572
Group^†^ (in Week 3)	1	0.1628	0.1307	0.1004
**Between-subject effects**
Group	1	0.1118	0.1807	0.1233
Error	4			
**Within-subject effects**
Week	2^‡^	**0.0026**	**0.0007**	**0.0006**
Week × Group	2	0.1718	**0.0035**	**0.0809**
Error (Week)	8			

^†^Except for N/A, the Error df is 4 (df: number of degrees of freedom).

^‡^Corn plants of the salt-stressed group had no upper root in Week 1. It follows: (i) the non-relevance of performing a statistical test to compare groups for their upper-root lengths in Week 1; (ii) within-subject effects with 1, 1, and 4 df instead of 2, 2, and 8 df, due to the inclusion of 2 weeks (Weeks 2 and 3) instead of the 3 weeks in the ANOVAR for upper-root length.

**Table 5:** Repeated measures analysis of variance results for length increments for corn lower- and upper-root systems and the two combined.

Source	*df*	Lower roots	*df*	Upper roots	*df*	Lower and upper roots combined
				***P*-value**		

Group^†^ (Weeks 1–2)	1	**0.0538**	N/A	N/A	1	0.1343
Group^†^ (Weeks 2–3)	1	0.4578	1	**0.0035**	1	0.2013
**Between-subject effects**
Group	1	0.1642			1	0.1018
Error	4				4	
**Within-subject effects**
Time	1	0.8859			1	0.2938
Time Group	1	0.4683			1	0.4935
Error (Time)	4				4	

^†^Except for N/A, the Error df is 4 (df: number of degrees of freedom).

^‡^Corn plants of the salt-stressed group had no upper root in Week 1, so length increments based on upper-root length could be calculated only from Weeks 2–3 for them, limiting the comparison between groups to a classical type of ANOVA (i.e., without repeated measures).

Given the above understanding of salinity stress on roots, and shoots, of corn, the CT scanning results that we presented add a new dimension to the understanding of root architecture patterns in growing corn seedlings, unstressed, and in salinity stress condition. This root repeated CT scanning experiment was to quantify and illustrate *in situ* the effects of salinity on germinating and growing corn seedlings at optimal growth temperature, as salinity stress is a very simple abiotic stress to simulate under laboratory conditions.

As possible future perspectives or studies, we can see and propose what follows on the basis of the results obtained in our study. Furthering this study to root growth patterns under cold stress will be very insightful because cold stress delays the onset of roots and the growth pattern is slower as compared to optimal conditions, thereby allowing CT scanning for two or three additional weeks using the experimental approach discussed in Subsection “Experimental Approach.” Slowing down the root growth can add to the refinement of the study of root branching patterns, volumes and lengths, and derived growth rates and increments. Such a study could be extended to other agriculturally important crops and their commercial genotypes, especially crops cultivated in temperate regions, with larger sample sizes.

## Conflict of Interest Statement

The authors declare that the research was conducted in the absence of any commercial or financial relationships that could be construed as a potential conflict of interest.
